# Numerical Modeling for Simulation of Compaction of Refractory Materials for Secondary Steelmaking

**DOI:** 10.3390/ma13010224

**Published:** 2020-01-04

**Authors:** Cristina Ramírez-Aragón, Joaquín Ordieres-Meré, Fernando Alba-Elías, Ana González-Marcos

**Affiliations:** 1 Department of Mechanical Engineering, University of La Rioja, C/Luis de Ulloa, 20, 26004 Logroño, Spain; maria-cristina.ramirez@alum.unirioja.es (C.R.-A.); fernando.alba@unirioja.es (F.A.-E.); ana.gonzalez@unirioja.es (A.G.-M.); 2 Department of Industrial Engineering, Business Administration and Statistics, Universidad Politécnica de Madrid, C/José Gutiérrez Abascal 2, 28006 Madrid, Spain

**Keywords:** powder compaction, refractory materials, discrete element method (DEM), experiments, cohesive contact models

## Abstract

The purpose of this work is to simulate the powder compaction of refractory materials, using the discrete element method (DEM). The capability of two cohesive contact models, implemented in different DEM packages, to simulate the compaction of a mixture of two refractory materials (dead burnt magnesia (MgO) and calcined alumina (Al_2_O_3_)) was analyzed, and the simulation results were compared with experimental data. The maximum force applied by the punch and the porosity and final shape quality of the compact were examined. As a starting point, the influence of Young’s modulus (E), the cohesion energy density (CED), and the diameter of the Al_2_O_3_ particles (D) on the results was analyzed. This analysis allowed to distinguish that E and CED were the most influential factors. Therefore, a more extensive examination of these two factors was performed afterward, using a fixed value of D. The analysis of the combined effect of these factors made it possible to calibrate the DEM models, and consequently, after this calibration, the compacts had an adequate final shape quality and the maximum force applied in the simulations matched with the experimental one. However, the porosity of the simulated compacts was higher than that of the real ones. To reduce the porosity of the compacts, lower values of D were also modeled. Consequently, the relative deviation of the porosity was reduced from 40–50% to 20%, using a value of D equal to 0.15 mm.

## 1. Introduction

Sintering is a processing technique that consists of compacting metal or ceramic powders to form solid components by applying thermal energy. This technique is interesting because it allows to obtain the parts of controlled porosity composed of different materials (combining distinct metals and/or ceramics). Moreover, the parts can be mass produced, and only a little amount of material is wasted during the process because the parts are produced with their final shapes.

Three processes are typically involved in the sintering technique: (1) powder synthesis, (2) compaction, and (3) heat treatment [1]. The first of them consists of the preparation of the powder blend (milling, blending, etc.). The second one consists of the compaction of the powder to form a part called “green body”. The last one consists of the application of heat treatment to the green body to obtain the sintered product. The properties of the final products are affected by all the processes involved in the sintering process. For example, some factors such as the firing temperature or dwell time must be carefully controlled during the heat treatment because they influence the physical and mechanical properties of the sintered parts [2]. Alternatively, some of the problems in the sintered parts are derived from the compaction process, including incorrect density, size or shape, variations in density throughout the parts, and cracking. Moreover, the raw materials influence the properties of the sintered parts. For example, the sizes and shapes of the particles affect the packing density or the porosity of the green compacts and, therefore, the pore sizes of the sintered parts [3,4,5]. As a consequence, the combination of different refractory materials has been also analyzed to improve the corrosion resistance of refractory parts by modifying their porosities [6,7,8].

The discrete element method (DEM) is a numerical method that is widely accepted as a useful tool to analyze the behavior of granular materials and solve different engineering problems, such as granular flows, powder mechanics, and rock mechanics [9]. Therefore, simulations using DEM have been used to analyze a great variety of processes, including the processes involving the sintering technique. The DEM simulations have been used to simulate the mill process [10,11], powder mixing [12,13], or powder packing [14,15,16,17,18,19,20,21,22,23,24]. Moreover, some approaches have been developed over the last years to simulate the behavior of the particles during the heat treatment in the sintering process [25,26,27,28,29]. In these works, the authors incorporated the concepts of the model developed by Parhami et al. [30] into three-dimensional (3D) DEM models. Usually, the contact model that describes the behavior of the particles under the heat treatment is combined with the other contact model that is used to compact the powder previously [29]. Therefore, it is interesting to acquire knowledge about the contact models that allow us to simulate the powder compaction because a better understanding of them would help in developing more realistic DEM simulations of the sintering process.

Different contact models and DEM packages have been used to simulate powder compaction. Janda and Ooi [14] used a visco-elasto-plastic frictional adhesive model to simulate confined compression and then unconfined compression and cone penetration resistant tests. Moreover, they investigated the possibility of calibrating the parameters of the contact model using big particles in the simulations to produce the same behavior instead of using small ones, thereby reducing the number of particles used and the computational cost required to complete the simulations. Using coarse particles and low values of Young’s modulus are some of the most usual approaches that researchers assume to reduce the computational cost. Thakur et al. [15] also used the API (application programming interface) in EDEM (DEM Solutions Ltd., Edinburgh, UK), to implement an adhesive elasto-plastic contact model to simulate cohesive powders under confined and unconfined compression. This contact model was used later [16] to investigate the scalability of the system, as done by Janda and Ooi [14]. Garner et al. [31] proposed another adhesive elasto-plastic contact model to simulate the powder compaction. The authors calibrated that model by the simulations of the compaction and ejection and tensile strength of the monosized particles. Yoon [17] used a bonded-particle model to simulate a uniaxial compression test. This type of contact model generates bonds between the particles that are in contact to keep them joined. The bond between two particles is broken when the stress between them exceeds a critical value. The effects of some microparameters of the bonded-particle model and other ones of the particles on the macroscopic responses of the materials under uniaxial compression were analyzed. The results allowed to develop a mathematical model for estimating the set of microparameters that must be set to simulate the behavior of a real material. Therefore, the bonded-particle model was calibrated and the results of the simulations were compared with the results of experiments that other authors had carried out previously using different types of rocks. Other authors combined the effect of a bonded-particle model with other contact models to simulate the behavior of spherical [18,19] and nonspherical [20,21] particles under confined and unconfined compression. In all the cases, they used the combination of an elasto-plastic model with other contact model that introduces capillary forces to simulate the powder compaction. After that, they applied a bonded-particle model to simulate the brittle failure of the compact. The effects of the particle size distribution [22] and the particle shape [23] on the mechanical responses of granular materials subjected to uniaxial compression were also studied by other authors. The effects of the particle size distribution were also analyzed by Nordström et al. [24], who implemented other contact models for this purpose. They simulated the compaction of several binary mixtures of different-sized particles and compared the results obtained with mixtures whose particle size ratios were 1:2 and 1:4. The results showed differences in most of the analyzed results: the relative density of the compacts, strain–pressure curves, coordination number, etc.

Because of the existence of different DEM packages and contact models, some authors have compared the results obtained using several of them. Jiménez-Herrera et al. [32] used two contact models implemented in EDEM and other one in ROCKY (ESSS, Florianópolis, Brazil) to simulate the breakage of particle beds when a ball hits them. Markauskas et al. compared the flow patterns, the velocity profiles, and discharge rates during the discharge of a silo obtained using EDEM and DEMMAT (swMATH, Berlin, Germany) [33]. Similarly, Wei et al. studied the charging of burden and the formation of burden layers in a blast furnace, using EDEM and LIGGGHTS (DCS Computing GmbH, Linz, Austria) [34]. Soltanbeigi et al. have also made a complete comparison between the results obtained with EDEM and LIGGGHTS, using nonspherical particles that include heap formation, direct shear test, and silo discharge [35]. In a previous work [36], three cohesive contact models implemented in different DEM simulators were used to examine their applicability to simulate the powder compaction. Several simulations were conducted for this purpose, which allowed to analyze the effects of some parameters on the results. Moreover, their results were compared and it was possible to determine that the “linear cohesion” model implemented in EDEM and the “SJKR2” model implemented in LIGGGHTS were equivalent contact models. However, in that work, the results of the simulations were not compared with experiments, and therefore, the capacity of those contact models to simulate the compaction of real materials were not examined.

In this work, the compaction of a refractory mixture to form a green compact was analyzed using DEM. Simulations were conducted using the equivalent contact models implemented in two DEM software packages that were determined previously [36]. A design of experiments (DOE) that comprehended 15 simulations was conducted to examine the influence of three parameters (Young’s modulus, cohesion energy density and particle size). After that, the effect of the two most influential parameters (Young’s modulus and cohesion energy density) was analyzed. Another DOE was applied for this purpose. This DOE also allowed to calibrate both simulation models. The results obtained using each software were compared to observe similarities and differences between them. The simulated results were also compared with experimental data in order to validate the DEM models. All of these simulations modeled particles larger than the real ones in order to reduce the computational costs during the analysis of the parameters and the calibration of the models. Finally, additional simulations were conducted using smaller particles to reduce the differences between the results of the simulations and the experimental ones.

The goal of this article is to promote a methodology enabling numerical simulation tools to help technical decision-makers in daily activities when the configuration of new materials to be built by sintering. Therefore, accuracy, computational cost, and time needs to be balanced. The computational cost is mainly influenced by the number of particles and the critical timestep. Moreover, this latter parameter is determined by the minimum particle size and the density and Young’s modulus of the material. Consequently, different strategies based on the modification of these four parameters have been used to reduce the computational cost [37,38,39]. In this work, the computational cost was reduced by means of one of those strategies that consists of simulating particles larger than the real ones, but keeping the real domain size [40], thereby leading to a greater critical timestep and reducing the number of particles. Special care was devoted to implementing this strategy but having a calibration procedure that makes it possible to keep the accuracy over a significant threshold. In this way, the procedure can be useful for practitioners because of the aforementioned balance accuracy versus resources.

## 2. Materials and Methods

### 2.1. Experimental Procedure 

**Figure 1:**
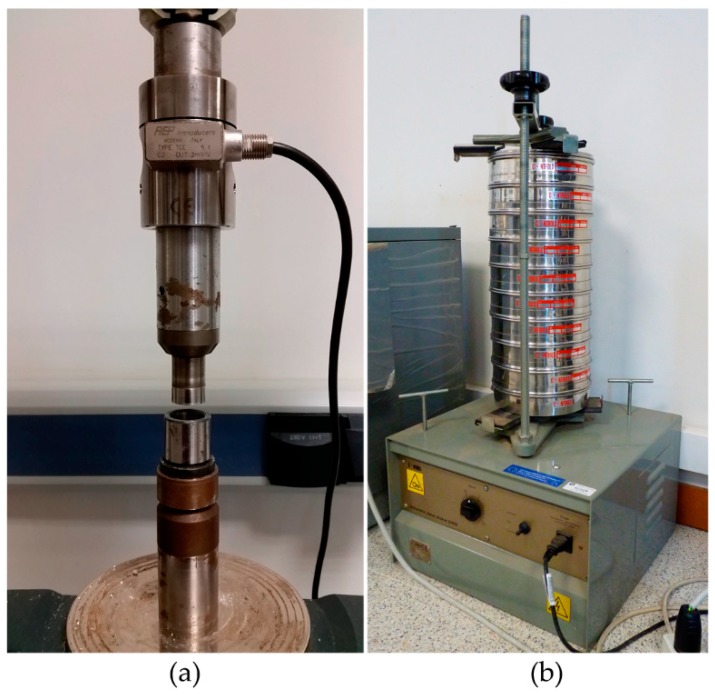
Equipment used in experiments. (**a**) Triaxial machine adapted to the compaction process. (**b**) Mechanical shaker with the sieves used for the gradation test.

A triaxial test machine (as shown in Figure 1a) has been adapted to compact the powder. It consists of the original frame and control system, a force sensor that measures the applied force by an upper punch, a lower punch, and a matrix. The diameter of the matrix was 22.05 mm. During this process, the speed of the punch was fixed and its displacement was calculated through time readings. Meanwhile, the force sensor measured the force applied by the punch. At the beginning of the process, the matrix and lower punch were assembled, thereby forming a cavity (die) with a diameter of 22.05 mm and height 11 mm. Then, the material (9.13 g in all) was introduced in the die. The upper punch was initially located at a distance of 20 mm over the lower punch. Once the machine and the material were ready, the upper punch began to move down, with a velocity of 1 mm/min. The movement of the upper punch stopped when the distance between the punches was 8 mm, and it began moving up, with a velocity of 25 mm/min, until it reached its initial position. Then, the matrix was removed and the compact was collected. 

**Figure 2:**
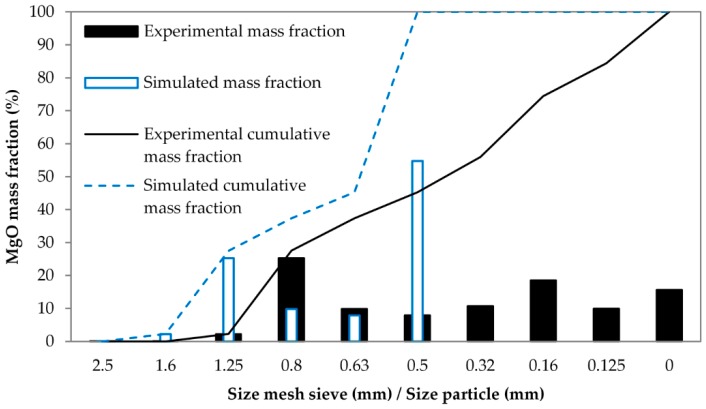
Results obtained in the gradation test for MgO and PSD used in simulations.

The materials used in this work included dead burnt magnesia (MgO) and calcined alumina (Al_2_O_3_). They were supplied by Refractory Solutions Insertec, S.L.U. These two materials are usually used for sintering of refractory castables [6,8,41,42]. The particle size of MgO was lower than 3 mm. A gradation test was performed to define its particle size distribution (PSD), which consists of introducing the granular material in a nested column of sieves with different-sized wire mesh. The sieves are orderly nested. The bigger mesh sieve is placed at the top and the smaller mesh sieve at the bottom of the column. A round pan (called “receiver”) is placed below the smaller mesh sieve. The nested column is placed in a mechanical shaker (see Figure 1b). This equipment shakes the column for several minutes. After that, the column is disassembled and the material on each sieve is weighed. Figure 2 presents the gradation test results for MgO. The particle size of Al_2_O_3_ was less than 45 µm. A mixture that consisted of Al_2_O_3_ and MgO in proportions of 20% and 80% (by weight), respectively, was used.

### 2.2. DEM Simulations

The DEM simulations were performed using two software packages: (1) EDEM^®^ 2.7.2, which is a commercial software (DEM Solutions Ltd., Edinburgh, UK) and (2) LIGGGHTS-PUBLIC^®^ 3.3.1, which is an open-source software (DCS ComputingGmbH, Linz, Austria). The “linear cohesion” contact model was employed in the simulations that ran in EDEM. The “modified simplified Johnson–Kendall–Roberts (SJKR2)” contact model was used in LIGGGHTS. In a previous work [36], the equivalence between these contact models was discussed. Both models work in combination with the Hertz–Mindlin contact model and complement it by the addition of a normal cohesion force. This normal cohesion force takes the form F_cohesion_ = k·A, where k is the cohesion energy density (J/m^3^) and A is the contact area (m^2^). This contact area takes the form A = 2·π·h·R, where h is the normal physical overlap between the particles (m) and R is the equivalent radius of the particles (m).

In order to simulate the experimental procedure, the geometries corresponding to the punches and matrix were modeled using a computer-aided design (CAD) software package (Solid Edge Version 18, © UGS Corp., Plano, TX, USA) and then converted into standard triangle language (STL) files. A tolerance of 0.01 mm was fixed to convert the 3D models into STL files. Finally, the same STL files were imported into both DEM simulators. In a previous work [36], the influence of using different conversion tolerances was analyzed. A value of 0.01 mm was considered optimum to convert similar geometries.

**Figure 3:**
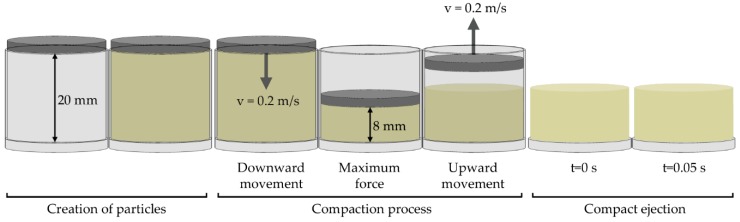
Scheme of the simulated process.

The simulation procedures are as follows: Initially, the particles were created in a virtual cylinder that had a height of 20 mm and a diameter of 22.05 mm. The amount of particles that was created in each simulation can be seen in Tables S1–S6 of the Supplementary material. Although the number of particles was slightly different in each simulation, approximately the same mass of mixture that was used in the experiments (9.13 g) was simulated in all cases. This factory was located in the space delimited by the inner surface of the matrix, lower surface of the upper punch, and upper surface of the lower punch. This volume was higher than the one adopted in experiments, where the height was 11 mm, in order to make the insertion of the particles possible in simulations. For that reason, the initial porosity of the material in the simulations was not compared with the initial porosity in the experiments. Once the particles had been created, the upper punch began to move down, with a constant velocity of 0.2 m/s. The downward movement stopped after 0.06 s. At this moment, the upper punch began to move up, with the same velocity during 0.04 s. Then, the matrix and upper punch disappeared in the simulation. Once the matrix had been removed, the simulation ran during 0.05 s in order to appreciate the final appearance of the compact. Figure 3 shows a scheme of the process that was simulated. A timestep equivalent to approximately 10% of the Rayleigh timestep was fixed in all simulations. In a previous work [36], this value was considered optimum for simulating the compaction process, using the same contact models that were used in this work. The effect of gravity force was taken into account in all simulations.

Some differences between simulations and experiments were assumed: Firstly, the particles did not settle at the beginning of the compaction in the simulations. However, a few minutes elapsed between the filling of the die and the compaction during the experimental testing, making the particles settle before the compaction started. This slightly affected the rearrangement of the particles in the simulations, but it greatly reduced the simulation time. Additional simulations were conducted to analyze this effect. The particles were settle in those simulations and the results of compaction were almost the same that those obtained when the particles were not settle. Secondly, the velocity of simulations was widely increased in order to reduce the computational cost. The influence of the speed of the punch was examined in a previous work [36], where a velocity of 0.1 m/s was considered adequate to simulate the compaction process, using the linear cohesion, SJKR, and SJKR2 models. This value was determined by the results obtained from the SJKR model. However, no differences in the results were found from 0.1 to 0.2 m/s for the linear cohesion and SJKR2 models, but the computational cost significantly reduced when a velocity of 0.2 m/s was used. For this reason, a velocity of 0.2 m/s was chosen in this work. Moreover, the velocity during the downward and upward movements was equal in the simulations, whereas the velocity in the upward movement was 25 times higher than that in the downward movement in the experiments. Finally, the simulations did not consider the effect of the walls during the ejection of the compact. The geometries relating to the matrix and upper punch were removed when the upper punch ended its upward movement, as mentioned above. This action was also conducted in other works [18,19]. 

The PSD used to simulate MgO was similar to the one obtained from the gradation test. However, the size of the simulated particles was decided to be higher than the real ones in order to reach a compromise between the hardware used and the simulation time, making it possible for practitioners to replicate the method. The PSD obtained from the gradation test and the simulated PSD are compared in Figure 2. The wide bars indicate the mass fraction of MgO that was retained in each sieve and in receiver (0 mm), and the narrow bars indicate the simulated PSD of MgO. As one can see, the experimental PSD was discretized and a minimum diameter was chosen for the MgO particles. This value was fixed to 0.5 mm. Therefore, the mass fraction corresponding to the real particles with diameters lower than 0.5 mm was simulated using particles with a diameter of 0.5 mm (scalping [37]). Similarly, the remaining amounts of material contained in each sieve were simulated with particles of diameters equal to the size of the mesh of the last sieve through which these materials passed (coarse graining [37]). This fact has been appreciated by a comparison of the experimental and simulated cumulative mass fraction lines provided in Figure 2. The particle size of Al_2_O_3_ was also greater in simulations. The simulated Al_2_O_3_ particles had diameters in the range of 0.3–0.5 mm in most of the setups of this work. These values are more than six times the real ones, but simulating particles of 45 µm would require an unacceptable computational cost. A few simulations using particles with a diameter of 0.15 mm were performed in order to reduce this difference. However, the computational cost of these latter simulations was approximately sixteen times the computational cost of their homologues with particles of diameter 0.3 mm. The properties of the materials that were simulated and their interaction parameters are summarized in Table 1 and Table 2, respectively. As one can see in these tables, the Young’s modulus of the materials and the cohesion energy density between particles were modified while other parameters kept constant. The relationships between the Young’s modulus of the different materials were equal in all the simulations. Similarly, the same value of cohesion energy density was chosen for all particle interactions in each simulation.

It should be noted that the values of the Young’s modulus of the simulated materials were lower than those of the real ones. However, this difference was assumed to reduce the computational cost, but also was necessary to counteract the effect of using greater particles than the real ones. The values of cohesion energy density were in the range 1 to 7 × 10^6^ J/m^3^ to avoid stability problems. This parameter is strongly dependent on some of the properties of the materials, such as the Young’s modulus and particle size and its calibration is necessary to accurately simulate the cohesive behavior of real materials [36]. Consequently, the range of values of cohesion energy density was stablished to ensure the stability of the simulations taking into account the range of Young’s modulus and particle sizes used in them.

### 2.3. Methodology

At the beginning of this work, several DEM models were performed. In these models, the compact process described in Section 2.2 was simulated in order to analyze the influence of Young’s modulus (E), the cohesion energy density (CED), and the particle size of Al_2_O_3_ (D) on the maximum force applied by the punch (F) and the porosity (P) and final shape quality of the compact (SQC). A DOE was applied for this purpose, where three levels of each factor (E, CED, and D) were examined. The details of the simulations used are provided in Table 3. Once these factors had been identified, another DOE was applied to verify if both software packages could generate results similar to the experimental data. For this purpose, three levels of the two most influential factors were examined, using both DEM packages. The simulations that were conducted are given in Table 4. The combined effect of these two factors was examined too. This analysis made it possible to predict the values of those factors that must be set to obtain results similar to the experimental ones in each DEM simulator. The simulations provided in Table 5 (setups nos. 1 and 2) were performed to validate this prediction. Finally, additional simulations using smaller Al_2_O_3_ particles were conducted to reduce the deviation between the experimental data and the results obtained from the simulations. The details of these simulations are presented in Table 5 (setups nos. 3−8).

As mentioned above, the maximum force applied by the punch and the porosity and final shape quality of the compacts were analyzed in simulations because of their importance in determining the goodness of the compacts: Firstly, some of the defects in the compacts, such as capping or lamination, are usually generated by the application of an inadequate force. The maximum force applied by the punch was chosen to compare the simulations with the experiments because the force was controlled in experimental testing and the comparison between experiments and simulations was immediate. The maximum force value was attained when the upper punch was located in its lower position in both cases. Secondly, the porosity of the compacts determine their mechanical properties. The porosity is the quotient between the volume of voids and the volume that is occupied by the compact (P = V_voids_/V_compact_). For simple geometries, such as the geometry of the compacts formed in this work, the porosity may be measured indirectly by determining the mass and dimensions of the component [43]. For this reason, the volume of the compact was calculated as the volume of a cylinder with the radius and the height of the compact (V_compact_ = π·r_compact_^2^ × h_compact_). The volume of the voids is the difference between the volume of the compact and the real volume of the material (V_voids_ = V_compact_ − V_material_). This volume is calculated in a different way in experiments and simulations. The real volume of the material was calculated experimentally as the summation of the volume of each material, which is calculated by the quotient between the mass and the density (V_material,exp_ = ∑m_i_/ρ_i_). Alternatively, the real volume of the simulated material was calculated as the difference between the summation of the volume of all the particles and the summation of the overlapped volume (V_material,sim_ = ∑V_particle_ − ∑V_overlap_). Lastly, the final SQC after the removal of the matrix was examined. The similarity ratio (or Kohonen similarity) taking into account the height of the compact and the average maximum radius at 15 levels of the height (from 0 to 0.015 m) just after the matrix had been removed and 0.05 s after that, was calculated and used to quantify this qualitative result (SQC). Moreover, a scale from 1 to 5 was applied to define the goodness of the compacts, where 5 means “very good SQC” and 1 means “very bad SQC.” The correspondence between each value of the scale and the Kohonen similarity was chosen taking into account that similarity ratios lower than 50% indicated “very bad SQC” and the optimal similarity ratio was 100%. The intermediate values of the scale were obtained by dividing the range between 50% and 100% in 4 equal parts. The scale used is the following: 5 ~ (100–87.5%); 4 ~ (87.5–75%); 3 ~ (75–62.5%); 2 ~ (62.5–50%); 1 ~ (50–0%).

## 3. Results and Discussion

### 3.1. Preliminary Analysis

The results obtained from the simulations that were conducted to determine the most influential parameters of the contact models are provided in Table 6. As one can see, the maximum force applied in experiments (25,095 N) was attained in the setups that used a Young’s modulus of 2500 MPa. Moreover, the porosity of the simulated compacts was greater than that of the real ones (26.08%) in all cases. Finally, good compacts were obtained in some cases but they were very bad in others, as the values of SQC indicated.

The results of the analysis of variance (ANOVA) of the quadratic model and the estimated regression coefficients for the maximum force, porosity of the compact, and shape quality of the compact can be seen in Tables S10–S12 of the Supplementary material, respectively. The DOE conducted, the levels of each factor and the results obtained are shown in Tables S7–S9. The regression models relating to the maximum force (Table S10) and porosity of the compact (Table S11) fitted the data well. The maximum force was mainly influenced by Young’s modulus in both DEM simulators. The regression models also indicated that the porosity of the compact increased with Young’s modulus and the diameter of the particles, but it decreased with CED. However, a good fitting between the regression models and the data was not possible for SQC, especially using EDEM (Table S12). Moreover, the ANOVA results revealed that Young’s modulus and CED were the factors that influenced, to a greater extent, all the results, using LIGGGHTS. However, the effect of the diameter was also important in EDEM, especially on the porosity of the compacts. Because the fitting for SQC using the data obtained from LIGGGHTS was better than that obtained from EDEM, the most influential parameters in LIGGGHTS were considered for the calibration of the models for both DEM simulators. Therefore, the effects of Young’s modulus and CED on the results have been covered in Section 3.2.

### 3.2. Calibration of the DEM Models

**Figure 4:**
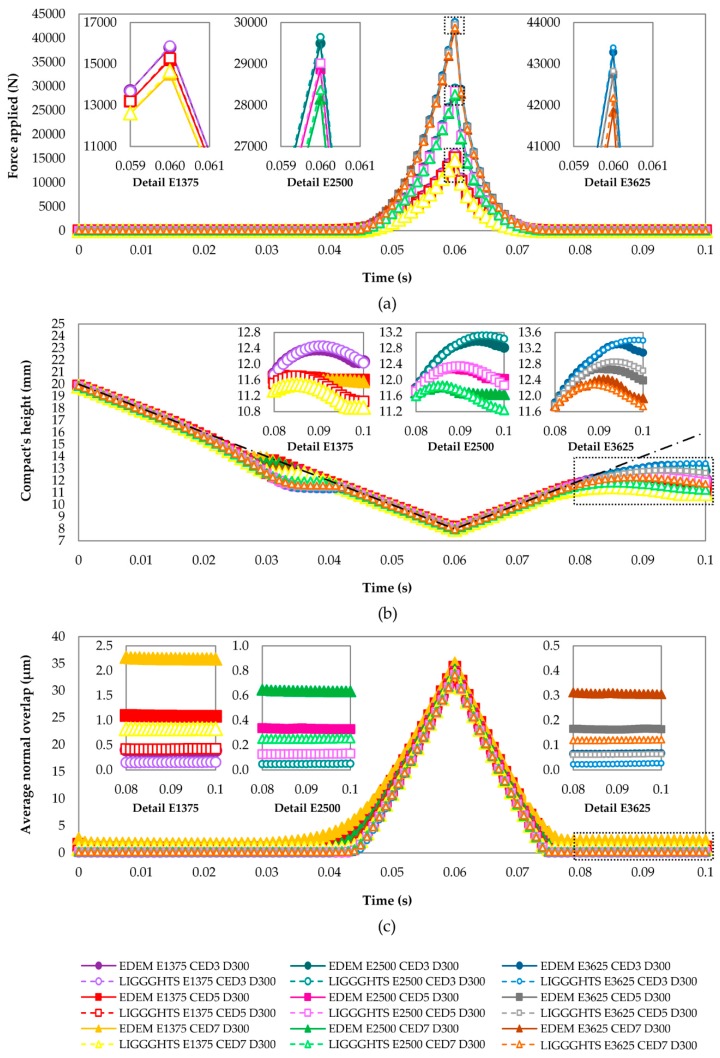
Results obtained in simulations relating to the DOE used to calibrate the DEM models. Evolution of (**a**) total force applied by the punch, (**b**) compact’s height and (**c**) average normal overlap between particles.

**Figure 5:**
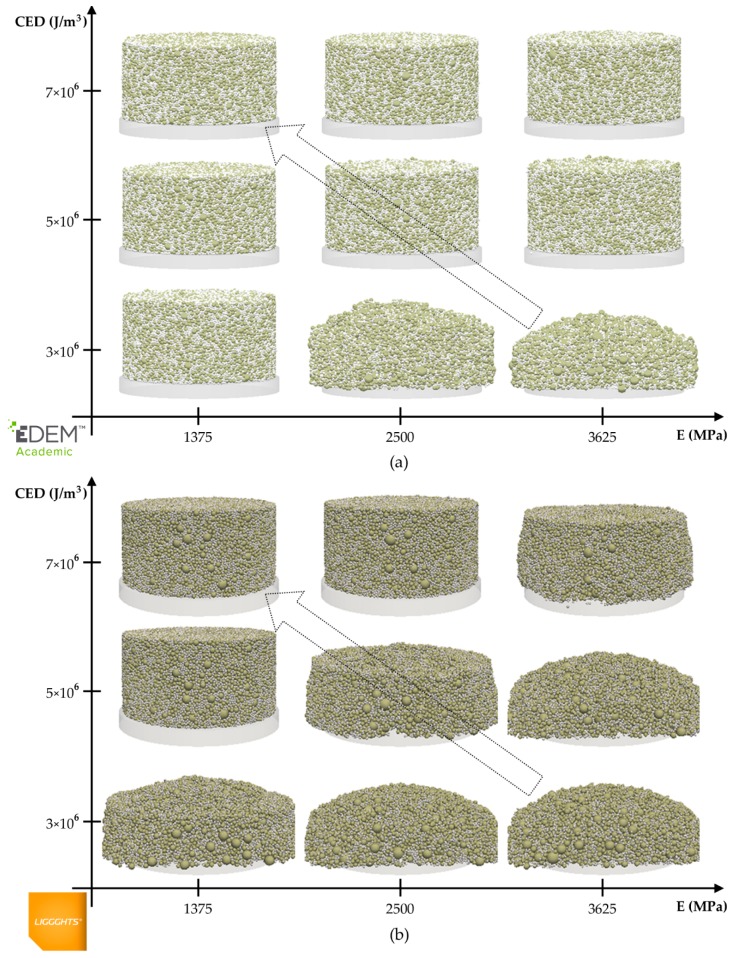
Results obtained in simulations relating to the DOE used to calibrate the DEM models. Final shape quality of the compacts obtained in (**a**) EDEM and (**b**) LIGGGHTS.

In this section, the effects of Young’s modulus and CED on the maximum force applied by the punch and the porosity and final shape quality of the compact are analyzed. Moreover, the analysis of the evolution of the height of the compact and overlap between the particles along the time supports the examination of the force. Figure 4 and Figure 5 show the results that were obtained from the nine simulations included in the DOE used to calibrate the DEM models (see Table 7). In addition, the regression models that were obtained from these simulations are presented in this section.

The evolution of the force applied by the punch along the time is shown in Figure 4a. Two sections are distinguished in this plot: The first of them corresponds to the loading process (from 0 to 0.06 s) and the second corresponds to the unloading process (from 0.06 to 0.1 s). The position of the punch along the time is represented by a black dot–dash line in Figure 4b. During the loading process, the punch moved down and the powder compaction took place. For this reason, the force increased until 0.06 s, when the maximum value of the force was attained. At this moment, the punch was located in its lowest position. After that, it began to move up. Consequently, the compact began to relax into its final dimensions. This induced the reduction of the force until its cessation. The cessation took place when the punch separated from the particles. As one can see in the detailed images (Figure 4a), the force reduced more quickly than it increased, although the velocity of the punch was the same during both processes (loading and unloading).

The evolution of the height of the compact along the time is shown in Figure 4b. It was calculated as the maximum distance between the particles along the z axis. In all cases, the curves showed some zones where the height of the compact matched the z position of the punch (represented by the black dot–dash line). Nevertheless, the height of the compact was lower than the z position of the punch in other zones. During the loading process (from 0 to 0.06 s), the height of the compact matched the z position of the punch at the beginning. This was because the particles were created in all the volume that was defined by the inner surfaces of the punches and matrix, and the upper punch began its movement just after the creation of the particles. For this reason, the punch pushed the particles down from this moment. The contact between the punch and particles kept for a brief moment. Then, the particles separated from the punch as a consequence of the impulse added by the punch. In this moment, the particles kept falling and then settled. The height of the compact was lower than the z position of the punch during this interval. After the settlement of the particles, the punch got in contact with the particles and began to push them down again until it stopped its downward movement, which resulted in a new matching between the height of the compact and the z position of the punch. During the unloading process (from 0.06 to 0.1 s), the compact initially expanded as the punch moved up because the contact forces between the particles were too high. Finally, the particles attained a steady state. For this reason, the height of the compact was relatively constant at the end of the process. Different behaviors took place at the end of this process in the simulations, as the detailed images of Figure 4b show. Although all the curves showed a curvature and reduction at the end of the process, some of them attained a constant value. This curvature was because some particles were taken off from the compact when the punch separated from the compact as a consequence of their interactions with the punch.

The average normal overlap between the particles along the time is shown in Figure 4c. As one can see, it was the greatest at the maximum load moment (0.06 s; see Figure 4a). Firstly, the particles had to overlap and be rearranged in order to fit in the volume delimited by the geometries, which made the volume to decrease as the upper punch moved down. The minimum volume took place at the maximum load moment (0.06 s) because the position of the upper punch was the lowest (8 mm; see Figure 4b). At this moment, the maximum values of normal overlap were attained. Secondly, the normal overlap was gradually reduced as the punch moved up at the beginning of the unloading process. This was due to the relaxation of the compact. Finally, the particles attained a steady state when the contact between the upper punch and particles disappeared (0.08 s, approximately). From this moment on, the normal overlap was kept relatively constant, as one can see in the detailed images of Figure 4c.

Despite the fact that the behavior of all the curves was generally the same, the effects of Young’s modulus and CED were identified: Firstly, three groups of curves were distinguished, as shown in Figure 4a. The curves corresponding to each level of Young’s modulus integrated each group. As expected, the Young’s modulus had a great influence on the maximum force applied by the punch. The force was higher as Young’s modulus was higher. These behaviors can be explained by means of an analysis of the average normal overlap between the particles (see Figure 4c). As one can see in the detailed images, the overlap was lower as Young’s modulus of the particles of a granular material was higher. This is because the deformations that occur at the contacts between the real particles are simulated by the overlap between the particles in the DEM models. The deformations of the real materials are lower as their Young’s modulus is higher. In the analyzed system, this increment of Young’s modulus induced that the granular material occupied a greater volume because the overlaps between the particles were lower. For this reason, the volume of the compact was also higher as Young’s modulus was higher. Because the transversal section was constant, the behavior of the heights of the compacts displayed in Figure 4b can explain the evolution of the volumes of the compacts along the time. Therefore, it was necessary to apply a higher force in order to reduce its volume (or height) until a determined value (8 mm in this case). Secondly, CED had a little influence on this maximum force (see Figure 4a). However, although the difference between the curves with equal Young’s modulus was too small, it was possible to appreciate that the maximum force decreased as CED increased (see the detailed images of Figure 4a). This was because the overlaps between the particles increased as a consequence of the attraction forces induced by the cohesion (as one can see in the detailed images of Figure 4c). The contact models used in this work considered that the cohesion force was proportional to the cohesion energy density. For this reason, as CED was higher, the overlap between the particles was higher too and the volume that they occupied was lower. The influence of CED on the final height of the compacts (at 0.1 s) is easy to perceive in Figure 4b. As one can see, the final height of the compacts was lower as CED was higher.

Figure 5 shows the final SQC 0.05 s after the matrix has been removed. As one can observe, the SQC depended on both parameters, Young’s modulus and CED. Although the shape qualities of the compacts that were simulated in EDEM did not match with their homologues in LIGGGHTS, it was possible to determine a similar trend in both simulators. The final SQC was better as Young’s modulus was lower and CED was higher.

Table 7 summarizes the results that were obtained from the simulations corresponding to the DOE used to calibrate the DEM models. As one can see, the maximum forces and porosity of the compacts were very similar in both software packages. Despite this similarity, the maximum forces applied by the punch in LIGGGHTS were slightly higher than the ones applied in EDEM. Moreover, the trends of both results were also similar for both DEM simulators. The maximum force increased considerably as Young’s modulus increased and decreased slightly as CED increased, as shown in Figure 4a. The porosity of the compacts behaved similar to the forces, but the effect of both parameters on the porosity was similar. This is in agreement with the results shown in Figure 4b. At 0.1 s, the height of the compact increases with Young’s modulus and decreases with CED. Because the porosity has been calculated as a function of the height of the compact in this work, both factors had the same effect on the porosity of the compact. However, the values assigned to the final SQC were different in some homologue simulations. Although the values obtained using one software did not match with the corresponding values obtained using the other, the trends in both software packages were the same (as shown in Figure 5). SQC got better as CED increased. However, the compacts tended to deteriorate as Young’s modulus increased. 

Furthermore, as one can see in Table 7, the force that was applied in the experiments (25095 N) is within the range of values obtained from the simulations. Therefore, it seems necessary to simulate a material with a Young’s modulus between 1375 and 2500 MPa. However, the porosity of the real compacts (26.08%) is out of range. This can be due to the approximation of the particle size distribution. The porosities of all the simulated compacts were higher than 35% because the sizes of the simulated particles were higher than those of the real ones. The porosity of the compacts can be reduced using smaller particles, as concluded in a preliminary analysis (see Section 3.1), but the computational cost would become too high. For this reason, it seems impossible to obtain a compact with a porosity of 26.08% by simulating the particles that were used in this work. Moreover, the appearance of the simulated compacts was good for those compacts with Young’s modulus of 1375 and 2500 MPa and CED of 7 × 106 J/m^3^. Therefore, it appears at first sight that it would be possible to obtain a good-shape-quality compact after the application of a similar force to the experimental one, although its porosity would be higher than the real one.

The ANOVA results of the quadratic model and the estimated regression coefficients for the maximum force, porosity of the compact, and shape quality of the compact can be seen in Tables S16–S18, respectively. The DOE conducted, the levels of each factor and the results obtained are shown in Tables S13–S15. As one can see, the regression predictions relating to the maximum force (Table S16) perfectly fitted the data. (R^2^ and adjusted R^2^ were equal to 1.) The regression models for the porosity of the compact (Table S17) also fitted the data well. In the case of SQC (Table S18), the fitting between the prediction and the data was worse, with R^2^ close to 0.9. Despite this, the regression model for EDEM relating to SQC was significantly better during the calibration than in the preliminary analysis (Table S12). 

Taking the ANOVA results into account, the regression models for the maximum force (F), porosity of the compact (P), and shape quality of the compact (SQC) using EDEM were determined by Equations (1)–(3), respectively. Equations (4)–(6) correspond to the regression predictions for LIGGGHTS as being dependent on Young’s modulus (E) and the cohesion energy density (CED).

F_EDEM_ = 28,900.822 + 13,729.317·E − 675.683·CED − 38.1·E·CED + 54.917·E^2^ − 92.683·CED^2^ (1)

P_EDEM_ = 41.34556 + 1.73833·E − 2.21167·CED − 0.6625·E·CED − 0.27833·E^2^ + 0.79167·CED^2^ (2)

SQC_EDEM_ = 5 − 0.33333·E + 0.5·CED + 0.5·E·CED − 0.5·CED^2^ (3)

F_LIGGGHTS_ = 29,026.111 + 13,763.667·E − 617·CED − 4.5·E·CED + 23.333E^2^ − 23.667·CED^2^ (4)

P_LIGGGHTS_ = 40.62111 + 3.07667·E − 3.68333·CED − 0.355·E·CED − 0.50667·E^2^ + 0.87333·CED^2^ (5)

SQC_LIGGGHTS_ = 3.77778 − 0.83333·E + 1.16667·CED − 0.16667·E^2^ − 0.16667·CED^2^ (6)

It must be noted that these equations were obtained for particles of Al_2_O_3_ with diameter equal to 0.3 mm, and therefore, the validity of these equations is only demonstrated for that diameter.

### 3.3. DEM Model Validation

Once the simulations conducted to calibrate the DEM models were completed, it was possible to predict the values that must be set in order to obtain results similar to the experimental ones, using each software. For this purpose, the values of the maximum force and porosity of the compact that were obtained in experimental testing and a value of 5 for SQC were used to resolve each one of the equation systems formed by Equations (1)–(3) and (4)–(6).

The parameters predicted and results obtained from the simulations corresponding to the validation of the models are provided in Table 8 (setup nos. 1 and 2). The parameters predicted in each software were the same, except for Young’s modulus. The value of this parameter in EDEM was slightly higher than that in LIGGGHTS because the maximum forces were lower in EDEM than in LIGGHTS for the same set of parameters (as mentioned in Section 3.2). Comparing the results obtained from both DEM simulators with the experimental ones, one can see that the simulated maximum force was closed from the experimental force. The deviations were lower than 60 N in both cases, and therefore, the relative difference was lower than 0.25% for both DEM models. However, the relative deviation between the experimental data and the results of the simulations was higher than 40% (49.2% EDEM; 41.2% LIGGGHTS) for the porosity. The simulated compacts had a porosity greater than 35%, whereas the porosity was 26.08%. This seems to indicate that the porosity of the real compact cannot be reached by simulating the Al_2_O_3_ material with particles of diameter 0.3 mm. Finally, SQC was “very good” in both simulations.

**Figure 6:**
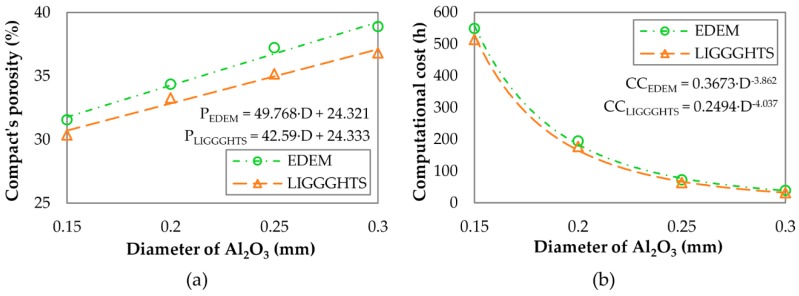
Effect of the particle size of Al2O3 on (**a**) the compact’s porosity and (**b**) the computational cost.

**Figure 7:**
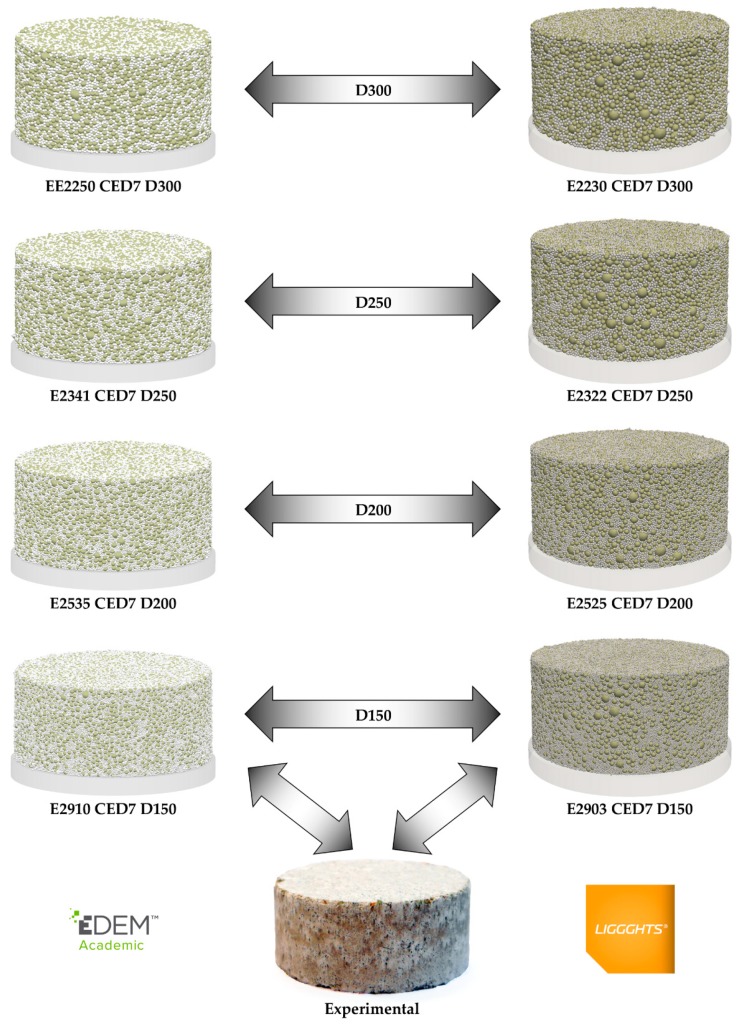
Green compacts obtained in the simulations used to validate the DEM models and real green compact.

In this work, some simplifications were assumed to reduce the computational cost during the calibration of the DEM models. The real PSD of MgO was discretized and the size of the Al_2_O_3_ particles was more than six times the size of the real ones. These actions reduced the width of the PSD of the mixture and, therefore, the packing density that might be attained [44]. For this reason, additional simulations using the Al_2_O_3_ particles with lower diameters (0.25, 0.2, and 0.15 mm) were modeled to check the ability of the simulations to form compacts with real porosities. The parameters used in these simulations are provided in Table 8 (setup nos. 3–8). The values of the maximum force applied by the punch were lower as the diameters were smaller, using the original Young’s modulus (2250 MPa in EDEM and 2230 MPa in LIGGGHTS). Because of this, Young’s modulus needed to be increased to compensate for the reduction of the maximum force that occurred when the size of the Al_2_O_3_ particles was reduced. In this work, only the results of the simulations with different-sized Al_2_O_3_ particles after their calibration have been presented. As provided in Table 8 (setups nos. 1–8), the values of the maximum forces were close to the experimental ones (25095 N). The relative deviation of the maximum forces was lower than 0.5% in all cases. On the contrary, the porosity was higher than the real one (26.08%) in all the cases. Nevertheless, one can appreciate that the porosity reduced as the particle size of Al_2_O_3_ was reduced, using both DEM simulators. The results show a reduction in the relative deviation of the porosity from 49.2% and 41.2% (with a diameter of 0.3 mm) to 21.0% and 16.4% (with a diameter of 0.15 mm) in EDEM and LIGGGHTS, respectively. Figure 6a plots the relationship between the porosity of the compact and the diameter of the Al_2_O_3_ particles. As one can see, the porosity decreases as the diameter of Al_2_O_3_ particles becomes smaller. The trend of the curve indicates that the real porosity would be reached if the particles with diameters similar to the real ones were simulated. The porosity of the compact using particles of diameter 0.045 mm is estimated to be 26.56% for EDEM and 26.25% for LIGGGHTS. These values indicate that the relative deviation would be lower than 2% in both simulators if these simulations were conducted. However, these simulations would require an unacceptable computational cost because the computational cost increases as the particle size of Al_2_O_3_ decreases, describing a power function as shown in Figure 6b. As a consequence, the minimum particle size simulated in this work was 0.15 mm. Each simulation that used particles of diameter 0.15 mm required approximately 3 weeks (515−550 h) to complete when they were run in conventional servers, which the practitioners usually have available. Additionally, the simulation conducted in LIGGGHTS with particles of diameter 0.15 mm was repeated using high-performance computing technology. This simulation was run under 64 cores and required approximately 30 h to complete. Although the reduction of the computational cost was important, lower diameters were not simulated because the technology required to keep the computational cost under acceptable figures is not commonly available to the practitioners and because the porosity reduction law becomes clearly inferred from the experiments that have already been carried out. As regards the appearance of the compacts after the matrix has been removed, the final SQC was 5 (“very good”) in all the simulations. The final appearance of the compacts simulated in both simulators using the Al_2_O_3_ particles of diameters 0.3, 0.25, 0.2, and 0.15 mm can be seen in Figure 7.

## 4. Conclusions

In this work, two DEM simulators, EDEM 2.7.2 and LIGGGHTS 3.3.1, were used to model the compaction of refractory materials. The contact models “linear cohesion” and “SJKR2”, implemented in EDEM and LIGGGHTS, respectively, were used for this purpose. The capability of these two contact models to simulate this process was examined by a comparison between the results of the simulations and those of the experiments. The maximum force applied by the punch and the porosity and final shape quality of the compact were the results that were considered in this work.

In order to analyze the capability of both models, it was necessary to calibrate them. Therefore, several simulations were conducted to study the effect of some parameters on the results. The parameters that were examined included Young’s modulus, CED, and the particle size of Al_2_O_3_. A preliminary analysis of these three factors determined that Young’s modulus and CED were the most influential factors. Therefore, the effects of these latter factors were examined in detail. The main conclusions that were obtained from these simulations are the following:

- The maximum force was mainly influenced by Young’s modulus. It increased significantly as Young’s modulus increased and decreased slightly as CED increased. The maximum force was also affected by the diameter of the Al_2_O_3_ particles, especially in EDEM.
- The porosity of the compacts increased with Young’s modulus and decreased with CED. The effect of both factors on the porosity was similar. Additionally, the porosity increased as the particle size of Al_2_O_3_ was larger. The effect of the diameter was also more remarkable in EDEM.
- SQC got better as CED increased, but it worsened with the increment of Young’s modulus. The effect of the particle size on SQC was negligible.
- The values of the maximum force and porosity of the compact were very similar in both software packages. On the contrary, there was not an equivalence between the values assigned to SQC in EDEM and LIGGGHTS. However, the trend in both software packages was the same.

By means of these simulations, it was also possible to obtain different regression models for each DEM simulator that allowed to predict the values of Young’s modulus and CED that were necessary to obtain simulated results similar to the real ones. The results obtained using both DEM packages were compared to experimental data to validate the DEM models. They showed that both DEM models were capable of reproducing the formation of the compacts after the application of a maximum force similar to the experimental one. However, the porosity of the simulated compacts was higher than that of the real ones. For this reason, additional simulations using the Al_2_O_3_ particles with lower diameters were conducted to reduce the porosity of the compacts. The relative deviation of the maximum force between the experiments and simulations was lower than 0.5% in all the simulations used to validate the DEM models. The relative deviation of the porosity was reduced from 40–50% to approximately 20% by means of the reduction in the particle size of Al_2_O_3_. SQC at the end of the simulations was “very good” in all of them.

In view of these results, the simulations using Al_2_O_3_ particles of diameter 0.3 mm may be useful to analyze some aspects of the powder compaction where the porosity of the compact is almost irrelevant—for example, the powder compaction to form green bodies with special shapes, including different sections, concave or convex surfaces. In other study cases where the porosity is critical, these simulations may be used to make a preliminary calibration of the model because of their low computational cost. After this preliminary calibration, other simulations should be conducted using a more realistic particle size distribution to validate the model.

The DEM models validated in this work would be used in future works to simulate the compaction of green bodies with more complex geometries. The distribution of the tension of the particles during the process and the possible inhomogeneities in the porosity of the compacts would be analyzed. Alternatively, the compaction of other mixtures consisting of different proportions of the materials used in this work would also be examined.

## Figures and Tables

**Table 1:** Summary of material properties used in DEM simulations.

Material Properties			
	MgO	Al_2_O_3_	Wall
Density (kg/m^3^)	3500	3000	8000
Young’s modulus (MPa)	250–3625	250–3625	200–2900
Poisson’s ratio	0.25	0.25	0.29

**Table 2:** Summary of interaction parameters used in DEM simulations.

Interaction Parameters			
	Particle-Particle	Particle-Wall	Wall-Wall
Coefficient of restitution	0.5	0.5	0.5
Coefficient of static friction	0.2	0.2	0.2
Coefficient of rolling friction	0	0	0
Cohesion energy density (J/m^3^)	1 × 10^6^–7 × 10^6^	–	–

**Table 3:** Setups used for the preliminary analysis of the DEM models.

Setup No.	Nomenclature	Young’s Modulus [MPa]	Cohesion Energy Density [J/m^3^]	Al_2_O_3_ Diameter [µm]
1	E0250 CED1 D300	250	1 × 10^6^	300
2	E0250 CED1 D500	250	1 × 10^6^	500
3	E0250 CED3 D400	250	3 × 10^6^	400
4	E0250 CED5 D300	250	5 × 10^6^	300
5	E0250 CED5 D500	250	5 × 10^6^	500
6	E1375 CED1 D400	1375	1 × 10^6^	400
7	E1375 CED3 D300	1375	3 × 10^6^	300
8	E1375 CED3 D400	1375	3 × 10^6^	400
9	E1375 CED3 D500	1375	3 × 10^6^	500
10	E1375 CED5 D400	1375	5 × 10^6^	400
11	E2500 CED1 D300	2500	1 × 10^6^	300
12	E2500 CED1 D500	2500	1 × 10^6^	500
13	E2500 CED3 D400	2500	3 × 10^6^	400
14	E2500 CED5 D300	2500	5 × 10^6^	300
15	E2500 CED5 D500	2500	5 × 10^6^	500

**Table 4:** Summary of interaction parameters used in DEM simulations.

Setup No.	Nomenclature	Young’s Modulus [MPa]	Cohesion Energy Density [J/m^3^]	Al_2_O_3_ Diameter [µm]
1	E1375 CED3 D300	1375	3 × 10^6^	300
2	E1375 CED5 D300	1375	5 × 10^6^	300
3	E1375 CED7 D300	1375	7 × 10^6^	300
4	E2500 CED3 D300	2500	3 × 10^6^	300
5	E2500 CED5 D300	2500	5 × 10^6^	300
6	E2500 CED7 D300	2500	7 × 10^6^	300
7	E3625 CED3 D300	3625	3 × 10^6^	300
8	E3625 CED5 D300	3625	5 × 10^6^	300
9	E3625 CED7 D300	3625	7 × 10^6^	300

**Table 5:** Setups used for the validation of the DEM models.

Setup No.	Nomenclature	DEM Simulator	Young’s Modulus [MPa]	Cohesion Energy Density [J/m^3^]	Al_2_O_3_ Diameter [µm]
1	E2250 CED7 D300	EDEM	2250	7 × 10^6^	300
2	E2230 CED7 D300	LIGGGHTS	2230	7 × 10^6^	300
3	E2341 CED7 D250	EDEM	2341	7 × 10^6^	250
4	E2322 CED7 D250	LIGGGHTS	2322	7 × 10^6^	250
5	E2535 CED7 D200	EDEM	2535	7 × 10^6^	200
6	E2525 CED7 D200	LIGGGHTS	2525	7 × 10^6^	200
7	E2910 CED7 D150	EDEM	2910	7 × 10^6^	150
8	E2903 CED7 D150	LIGGGHTS	2903	7 × 10^6^	150

**Table 6:** Results obtained in the simulations used for the preliminary analysis of the DEM models.

		EDEM			LIGGGHTS		
Setup No.	Nomenclature	F [N]	P [%]	SQC [-]	F [N]	P [%]	SQC [-]
1	E0250 CED1 D300	2729	41.86	5	2750	38.25	4
2	E0250 CED1 D500	2333	54.20	5	2919	41.90	4
3	E0250 CED3 D400	2260	48.02	5	2250	35.60	5
4	E0250 CED5 D300	1424	32.81	1	1457	29.37	5
5	E0250 CED5 D500	1182	45.21	5	1628	31.66	5
6	E1375 CED1 D400	17,091	55.29	2	17,276	47.95	2
7	E1375 CED3 D300	15,787	41.48	5	15,865	41.34	3
8	E1375 CED3 D400	16,551	51.24	5	16,624	43.47	3
9	E1375 CED3 D500	12,475	55.13	5	16,858	44.41	3
10	E1375 CED5 D400	15,922	48.94	5	15,986	38.24	5
11	E2500 CED1 D300	30,093	47.07	2	30,297	47.59	2
12	E2500 CED1 D500	23,613	58.17	2	31,987	48.94	2
13	E2500 CED3 D400	29,909	54.22	3	31,053	47.35	2
14	E2500 CED5 D300	28,867	41.32	5	29,015	40.51	4
15	E2500 CED5 D500	22,453	54.14	5	30,807	43.98	3

F: maximum force; P: compact’s porosity; SQC: final shape quality of the compacts.

**Table 7:** Results obtained in the simulations used for the calibration of the DEM models.

		EDEM			LIGGGHTS		
Setup no.	Nomenclature	F [N]	P [%]	SQC [-]	F [N]	P [%]	SQC [-]
1	E1375 CED3 D300	15,787	41.48	5	15,865	41.34	3
2	E1375 CED5 D300	15,201	39.14	5	15,283	36.20	5
3	E1375 CED7 D300	14,506	38.95	5	14,662	35.32	5
4	E2500 CED3 D300	29,495	44.93	4	29,647	45.87	2
5	E2500 CED5 D300	28,867	41.32	5	29,015	40.51	4
6	E2500 CED7 D300	28,155	39.37	5	28,369	37.23	5
7	E3625 CED3 D300	43,279	46.08	3	43,393	47.31	2
8	E3625 CED5 D300	42,745	43.02	5	42,827	44.14	2
9	E3625 CED7 D300	41,846	40.90	5	42,172	39.87	4

F: maximum force; P: compact’s porosity; SQC: final shape quality of the compacts.

**Table 8:** Results obtained in the simulations used for the validation of the DEM models.

Setup No.	Nomenclature	DEM Simulator	F [N]	P [%]	SQC [-]
1	E2250 CED7 D300	EDEM	25,153	38.90	5
2	E2230 CED7 D300	LIGGGHTS	25,134	36.83	5
3	E2341 CED7 D250	EDEM	25,089	37.24	5
4	E2322 CED7 D250	LIGGGHTS	25,075	35.19	5
5	E2535 CED7 D200	EDEM	24,970	34.37	5
6	E2525 CED7 D200	LIGGGHTS	25,012	33.28	5
7	E2910 CED7 D150	EDEM	25,159	31.57	5
8	E2903 CED7 D150	LIGGGHTS	25,135	30.37	5

F: maximum force; P: compact’s porosity; SQC: final shape quality of the compacts.

## Supplementary Materials

The following are available online at https://www.mdpi.com/1996-1944/13/1/224/s1.
