# Supplementary material for: Numerical Modeling for Simulation of Compaction of Refractory Materials for Secondary Steelmaking
Source: Materials (Basel). 2020 Jan 4;13(1):224. doi: 10.3390/ma13010224 (PMC6982119; doi:10.3390/ma13010224)
Supplement: Supplementary file 1 [file materials-13-00224-s001.pdf]

materials

Supplementary Materials

Numerical Modeling for Simulation of Compaction of Refractory Materials for Secondary Steelmaking

Cristina Ramírez-Aragón [1], Joaquín Ordieres-Meré [2,*], Fernando Alba-Elías [1] and Ana González-Marcos [1]

[1] Department of Mechanical Engineering, University of La Rioja, C/Luis de Ulloa, 20, 26004 Logroño, Spain; maria-cristina.ramirez@alum.unirioja.es (C.R.-A.); fernando.alba@unirioja.es (F.A.-E.); ana.gonzalez@unirioja.es (A.G.-M.)
[2] Department of Industrial Engineering, Business Administration and Statistics, Universidad Politécnica de Madrid, C/José Gutiérrez Abascal 2, 28006 Madrid, Spain
* Correspondence: j.ordieres@upm.es; Orcid: 0000-0002-9677-6764; Tel.: +34-91-067-7107

Received: 19 November 2019; Accepted: 2 January 2020; Published: 4 January 2020

S1. Number of Particles Simulated

Table S1. Number of particles simulated in the configurations that used Al_2O_3 particles of diameter 0.5 mm.

Material	Number of Particles	Particle Size (mm)
MgO	17368	0.5
	1252	0.63
	763	0.8
	504	1.25
	22	1.6
	2	2.5
Al_2O_3	9272	0.5

Table S2. Number of particles simulated in the configurations that used Al_2O_3 particles of diameter 0.4 mm.

Material	Number of Particles	Particle Size (mm)
MgO	17368	0.5
	1252	0.63
	763	0.8
	504	1.25
	24	1.6
	2	2.5
Al_2O_3	18111	0.4

Table S3. Number of particles simulated in the configurations that used Al_2O_3 particles of diameter 0.3 mm.

Material	Number of Particles	Particle Size (mm)
MgO	17368	0.5
	1252	0.63
	763	0.8
	504	1.25
	24	1.6
	2	2.5
Al_2O_3	42930	0.3

Table S4. Number of particles simulated in the configurations that used Al_2O_3 particles of diameter 0.25 mm.

Material	Number of Particles	Particle Size (mm)
MgO	17368	0.5
	1251	0.63
	763	0.8
	504	1.25
	25	1.6
	2	2.5
Al_2O_3	74183	0.25

Table S5. Number of particles simulated in the configurations that used Al_2O_3 particles of diameter 0.2 mm.

Material	Number of Particles	Particle Size (mm)
MgO	17368	0.5
	1252	0.63
	763	0.8
	504	1.25
	24	1.6
	2	2.5
Al_2O_3	144889	0.2

Table S6. Number of particles simulated in the configurations that used Al_2O_3 particles of diameter 0.15 mm.

Material	Number of Particles	Particle Size (mm)
MgO	17368	0.5
	1252	0.63
	763	0.8
	504	1.25
	24	1.6
	2	2.5
Al_2O_3	343441	0.15

S2. Combined Effect of the Young's Modulus (E), the Cohesion Energy Density (CED) and the Al_2O_3 Particle Size (D)

Table S7. Factors and levels used in the simulations conducted in the preliminary analysis of the DEM models.

Factor		Uncoded Value Assigned to Each Level			Transformation from Coded to Uncoded
		−1	0	1	
E:	Young's modulus [MPa]	250	1375	2500	×1 ~ (E - 1375)/1125
CED:	Cohesion energy density [J/m³]	1 × 10⁶	3 × 10⁶	5 × 10⁶	×2 ~ (CED—3 × 10⁶)/2 × 10⁶
D:	Particle size Al_2O_3 [μm]	300	400	500	×3 ~ (D-400)/100

Table S8. Setups of the simulations used in the preliminary analysis of the DEM models.

Setup Number	Nomenclature	Uncoded Values			Coded Values		
		E	CED	D	×1	×2	×3
1	E0250 CED1 D300	250	1×10^6	300	−1	−1	−1
2	E0250 CED1 D500	250	1×10^6	500	−1	−1	1
3	E0250 CED3 D400	250	3×10^6	400	−1	0	0
4	E0250 CED5 D300	250	5×10^6	300	−1	1	−1
5	E0250 CED5 D500	250	5×10^6	500	−1	1	1
6	E1375 CED1 D400	1375	1×10^6	400	0	−1	0
7	E1375 CED3 D300	1375	3×10^6	300	0	0	−1
8	E1375 CED3 D400	1375	3×10^6	400	0	0	0
9	E1375 CED3 D500	1375	3×10^6	500	0	0	1
10	E1375 CED5 D400	1375	5×10^6	400	0	1	0
11	E2500 CED1 D300	2500	1×10^6	300	1	−1	−1
12	E2500 CED1 D500	2500	1×10^6	500	1	−1	1
13	E2500 CED3 D400	2500	3×10^6	400	1	0	0
14	E2500 CED5 D300	2500	5×10^6	300	1	1	−1
15	E2500 CED5 D500	2500	5×10^6	500	1	1	1

Table S9. Results obtained in the simulations used in the preliminary analysis of the DEM models.

Setup Number	Nomenclature	EDEM			LIGGGHTS		
		Maximum Force [N]	Compact's Porosity [%]	Final Compact's Appearance	Maximum Force [N]	Compact's Porosity [%]	Final Compact's Appearance
1	E0250 CED1 D300	2729	41.86	5	2750	38.25	4
2	E0250 CED1 D500	2333	54.20	5	2919	41.90	4

3	E0250 CED3 D400	2260	48.02	5	2250	35.60	5
4	E0250 CED5 D300	1424	32.81	1	1457	29.37	5
5	E0250 CED5 D500	1182	45.21	5	1628	31.66	5
6	E1375 CED1 D400	17091	55.29	2	17276	47.95	2
7	E1375 CED3 D300	15787	41.48	5	15865	41.34	3
8	E1375 CED3 D400	16551	51.24	5	16624	43.47	3
9	E1375 CED3 D500	12475	55.13	5	16858	44.41	3
10	E1375 CED5 D400	15922	48.94	5	15986	38.24	5
11	E2500 CED1 D300	30093	47.07	2	30297	47.59	2
12	E2500 CED1 D500	23613	58.17	2	31987	48.94	2
13	E2500 CED3 D400	29909	54.22	3	31053	47.35	2
14	E2500 CED5 D300	28867	41.32	5	29015	40.51	4
15	E2500 CED5 D500	22453	54.14	5	30807	43.98	3

Table S10. ANOVA results of the quadratic model and estimated regression coefficients for MAXIMUM FORCE (Preliminary analysis).

EDEM						LIGGGHTS					
Source	DF	Sum of Squares	Mean Squares	F-value	*p*-value	Source	DF	Sum of Squares	Mean Squares	F-value	*p*-value
Model	9	1,630,498,613	181,166,512.56	204.828	<0.001	Model	9	2028635369	225,403,929.89	12,678.813	<0.001
Linear	3	1,594,630,358	531,543,453.00	600.966	<0.001	Linear	3	2027137315	675,712,438.00	38,007.960	<0.001
Square	3	18,784,905	6,261,635	7.0794	0.0300	Square	3	1237233	412411	23.1976	0.0023
Interaction	3	17,083,350	5,694,450	6.4382	0.0361	Interaction	3	260821	86940	4.8903	0.0600
Residuals	5	4,422,407	884,481	–	–	Residuals	5	88891	17778	–	–
Lack-of-fit	5	4,422,407	884,481	1	0.5	Lack-of-fit	5	88891	17778	1	0.5
Pure error	0	0	0	–	–	Pure error	–	0	0	–	–
Total	14	1,634,921,020	–	–	–	Total	14	2028724260	–	–	–
R^2 = 0.9973		–	–	–	–	R^2 = 1		–	–	–	–

Adj-R^2 = 0.9924	–	–	–	–	Adj-R^2 = 0.99990	–	–	–	–
**Term**	**Estimate**	**Std. Error**	**t-value**	***p*-value**	**Term**	**Estimate**	**Std. Error**	**t-value**	***p*-value**
Intercept	16,384.826	505.487	32.414	<0.001	Intercept	16,637.689	71.665	232.1581	<0.001
×1	12,500.613	297.402	42.0327	<0.001	×1	14215.5	42.164	337.1464	<0.001
×2	−600.972	297.402	−2.0207	0.0993	×2	-633.6	42.164	−15.027	<0.001
×3	−1684.204	297.402	−5.663	0.0024	×3	481.5	42.164	11.4196	<0.001
×1×2	8.865	332.506	0.0267	0.9798	×1×2	15.25	47.141	0.3235	0.7594
×1×3	−1532.082	332.506	−4.6077	0.0058	×1×3	392.75	47.141	8.3314	<0.001
×2×3	27.527	332.506	0.0828	0.9372	×2×3	13	47.141	0.2758	0.7938
$\times 1^2$	−259.047	586.485	−0.4417	0.6772	$\times 1^2$	10.389	83.149	0.1249	0.9054
$\times 2^2$	163.118	586.485	0.2781	0.7920	$\times 2^2$	−10.111	83.149	−0.1216	0.9079
$\times 3^2$	−2212.532	586.485	−3.7725	0.0130	$\times 3^2$	−279.611	83.149	−3.3628	0.0200

×1 ~ (E - 1375)/1125; ×2 ~ (CED - 3·× 10^6)/2·× 10^6; ×3 ~ (D-400)/100

Table S11. ANOVA results of the quadratic model and estimated regression coefficients for COMPACT's POROSITY (Preliminary analysis).

EDEM						LIGGGHTS					
**Source**	**DF**	**Sum of Squares**	**Mean Squares**	**F-value**	***p*-value**	**Source**	**DF**	**Sum of Squares**	**Mean Squares**	**F-value**	***p*-value**
Model	9	689.86	76.65	164.841	<0.001	Model	9	483.31	53.70	61.939	<0.001
Linear	3	612.61	204.202	439.301	<0.001	Linear	3	452.32	150.77	173.814	<0.001
Square	3	9.03	3.011	6.4766	0.0357	Square	3	6.49	2.165	2.4958	0.17433
Interaction	3	68.22	22.740	48.9206	0.0004	Interaction	3	24.5	8.165	9.4133	0.01689
Residuals	5	2.32	0.465	–	–	Residuals	5	4.34	0.867	–	–
Lack-of-fit	5	2.62	0.465	1	0.5	Lack-of-fit	5	4.34	0.867	1	0.5
Pure error	0	0	0	–	–	Pure error	0	0	0	–	–
Total	14	694.8	–	–	–	Total	14	491.99	–	–	–
R^2 = 0.9966		–	–	–	–	R^2 = 0.9911		–	–	–	–
Adj-R^2 = 0.9906		–	–	–	–	Adj-R^2 = 0.9751		–	–	–	–
**Term**		**Estimate**	**Std. Error**	**t-value**	***p*-value**	**Term**		**Estimate**	**Std. Error**	**t-value**	***p*-value**
Intercept		52.02622	0.36645	141.9738	<0.001	Intercept		43.55178	0.50059	87.0007	<0.001
×1		3.281	0.21560	15.2180	<0.001	×1		5.159	0.29452	17.5165	<0.001
×2		−3.418	0.21560	−15.8534	<0.001	×2		−4.087	0.29452	−13.8767	<0.001
×3		6.23	0.21560	28.8961	<0.001	×3		1.383	0.29452	4.6957	0.0054
×1×2		1.03375	0.24105	4.2886	0.0078	×1×2		0.885	0.32929	2.6876	0.0434
×1×3		−0.10125	0.24105	−0.4200	0.6919	×1×3		−0.14	0.32929	−0.4252	0.6884
×2×3		0.22375	0.24105	0.9282	0.3959	×2×3		0.095	0.32929	0.2885	0.7845
$\times 1^2$		−1.10278	0.42517	−2.5937	0.0486	$\times 1^2$		−2.09722	0.58081	−3.6109	0.0154
$\times 2^2$		−0.10778	0.42517	−0.2535	0.8100	$\times 2^2$		−0.47722	0.58081	−0.8217	0.4487
$\times 3^2$		−3.91778	0.42517	−9.2146	<0.001	$\times 3^2$		−0.69722	0.58081	−1.2004	0.2837

×1 ~ (E - 1375)/1125; ×2 ~ (CED - 3·× 10^6)/2·× 10^6;·×3 ~ (D-400)/100

Table S12. ANOVA results of the quadratic model and estimated regression coefficients for SHAPE QUALITY of the COMPACT (Preliminary analysis).

EDEM							LIGGGHTS					
**Source**	**DF**	**Sum of Squares**	**Mean Squares**	**F-value**	***p*-value**		**Source**	**DF**	**Sum of Squares**	**Mean Squares**	**F-value**	***p*-value**
Model	9	25.9222	2.88	2.369	0.1775		Model	9	17.7083	1.97	4.858	0.0483
Linear	3	5.7	1.90	1.563	0.30854		Linear	3	16.5	5.50	13.580	0.0077
Square	3	16.5	5.5	4.5247	0.06879		Square	3	0.375	1.25	0.3086	0.8189
Interaction	3	3.7222	1.2407	1.0207	0.45745		Interaction	3	0.8333	0.2778	0.5859	0.5982
Residuals	5	6.0778	1.2156	–	–		Residuals	5	2.025	0.405	–	–
Lack-of-fit	5	6.0778	1.2156	1	0.5		Lack-of-fit	5	2.025	0.405	1	0.5
Pure error	0	0	0	–	–		Pure error	0	0	1	–	–
Total	14	32	–	–	–		Total	14	19.7333	–	–	–
R^2 = 0.8101		–	–	–	–		R^2 = 0.8974		–	–	–	–
Adj-R^2 = 0.4682		–	–	–	–		Adj-R^2 = 0.7127		–	–	–	–

EDEM						LIGGGHTS				
**Term**	**Estimate**	**Std. Error**	**t-value**	***p*-value**		**Term**	**Estimate**	**Std. Error**	**t-value**	***p*-value**
Intercept	4.55556	0.59259	7.6876	<0.001		Intercept	3.13333	0.34205	9.1604	<0.001
×1	−0.4	0.34865	−1.1473	0.3032		×1	−1	0.20125	−4.9690	0.0042
×2	0.5	0.34865	1.4341	0.2110		×2	0.8	0.20125	3.9752	0.0106
×3	0.4	0.34865	1.1473	0.3032		×3	−0.1	0.20125	−0.4969	0.6403
×1×2	1.25	0.38980	3.2068	0.0238		×1×2	0.125	0.22500	0.5556	0.6025
×1×3	−0.5	0.38980	−1.2827	0.2558		×1×3	−0.125	0.22500	−0.5556	0.6025
×2×3	0.5	0.38980	1.2827	0.2558		×2×3	−0.125	0.22500	−0.5556	0.6025
×1^2	−0.44444	0.68754	−0.6464	0.5465		×1^2	0.33333	0.39686	0.8399	0.4393
×2^2	−0.94444	0.68754	−1.3737	0.2279		×2^2	0.33333	0.39686	0.8399	0.4393
×3^2	0.55556	0.68754	0.8080	0.4558		×3^2	−0.16667	0.39686	−0.4200	0.6920

×1 ~ (E - 1375)/1125; ×2 ~ (CED - 3·× 10^6)/2·× 10^6; ×3 ~ (D-400)/100

S3. Combined Effect of the Young's Modulus (E) and the Cohesion Energy Density (CED)

Table S13. Factors and levels used in the simulations conducted in the calibration of the DEM models.

**Factor**		**Uncoded Value Assigned to Each Level**			**Transformation from Coded to Uncoded**
		**−1**	**0**	**1**	
E:	Young's modulus [MPa]	1375	2500	3625	×1 ~ (E - 2500)/1125
CED:	Cohesion energy density [J/m^3]	3·× 10^6	5·× 10^6	7·× 10^6	×2 ~ (CED - 5·× 10^6)/2·× 10^6

Table S14. Setups of the simulations used to calibrate of the DEM models.

Setup Number	Nomenclature	Uncoded Values		Coded Values	
		E	CED	×1	×2
1	E1375 CED3 D300	1375	$3{\cdot}\times 10^6$	−1	−1
2	E1375 CED5 D300	1375	$5{\cdot}\times 10^6$	−1	0
3	E1375 CED7 D300	1375	$7{\cdot}\times 10^6$	−1	1
4	E2500 CED3 D300	2500	$3{\cdot}\times 10^6$	0	−1
5	E2500 CED5 D300	2500	$5{\cdot}\times 10^6$	0	0
6	E2500 CED7 D300	2500	$7{\cdot}\times 10^6$	0	1
7	E3625 CED3 D300	3625	$3{\cdot}\times 10^6$	1	−1
8	E3625 CED5 D300	3625	$5{\cdot}\times 10^6$	1	0
9	E3625 CED7 D300	3625	$7{\cdot}\times 10^6$	1	1

Table S15. Results obtained in the simulations used to calibrate of the DEM models.

Setup Number	Nomenclature	EDEM			LIGGGHTS		
		Maximum Force [N]	Compact's Porosity [%]	Final Compact's Appearance	Maximum Force [N]	Compact's Porosity [%]	Final Compact's Appearance
1	E1375 CED3 D300	15787	41.48	5	15865	41.34	2
2	E1375 CED5 D300	15201	39.14	5	15283	36.20	5
3	E1375 CED7 D300	14506	38.94	5	14662	35.31	5
4	E2500 CED3 D300	29495	44.93	2	29647	45.87	1
5	E2500 CED5 D300	28867	41.32	5	29015	40.51	3
6	E2500 CED7 D300	28155	39.37	5	28369	37.23	5
7	E3625 CED3 D300	43279	46.08	2	43393	47.31	1
8	E3625 CED5 D300	42745	43.02	5	42827	44.14	2
9	E3625 CED7 D300	41846	40.90	5	42172	39.87	3

Table S16. ANOVA results of the quadratic model and estimated regression coefficients for MAXIMUM FORCE (Calibration).

EDEM						LIGGGHTS					
**Source**	**DF**	**Sum of Squares**	**Mean Squares**	**F-value**	***p*-value**	**Source**	**DF**	**Sum of Squares**	**Mean Squares**	**F-value**	***p*-value**
Model	5	1,133,733,123	226,746,624.60	83118.264	<0.001	Model	5	1,138,917,545	227,783,509.00	352,606.051	<0.001
Linear	2	1,133,704,105	566,852,052.00	2.08×10^5	<0.001	Linear	2	1,138,915,255	569,457,627.00	8.81×10^5	<0.001
Square	1	5806	5806	2.13	0.2407	Square	1	81	81	0.125	0.7467
Interaction	2	23212	11606	4.25	0.1331	Interaction	2	2209	1105	1.71	0.3195
Residuals	3	8185	2728	–	–	Residuals	3	1938	646	–	–
Lack-of-fit	3	8185	2728	1	0.5	Lack-of-fit	3	1938	646	1	0.5
Pure error	0	0	0	–	–	Pure error	0	0	0	–	–
Total	11	1133741308	–	–	–	Total	11	1138919483	–	–	–
$R^2 = 1$		–	–	–	–	$R^2 = 1$		–	–	–	–
Adj-$R^2 = 1$		–	–	–	–	Adj-$R^2 = 1$		–	–	–	–
**Term**		**Estimate**	**Std. Error**	**t-value**	***p*-value**	**Term**		**Estimate**	**Std. Error**	**t-value**	***p*-value**
Intercept		28900.822	38.932	742.3359	<0.001	Intercept		29026.111	18.945	1532.1328	<0.001
×1		13729.317	21.324	643.8409	<0.001	×1		13763.667	10.377	1326.42	<0.001
×2		−675.683	21.324	−31.6864	<0.001	×2		−617	10.377	−59.461	<0.001
×1×2		−38.1	26.117	−1.4588	0.2407	×1×2		−4.5	12.709	−0.3541	0.7467
$\times1^2$		54.917	36.934	1.4869	0.2338	$\times1^2$		23.333	17.973	1.2983	0.2850
$\times2^2$		−92.683	36.934	−2.5094	0.0870	$\times2^2$		−23.667	17.973	−1.3168	0.2795

×1 ~ (E - 2500)/1125; ×2 ~ (CED - 5×10^6)/2×10^6

Table S17. ANOVA results of the quadratic model and estimated regression coefficients for COMPACT's POROSITY (Calibration).

EDEM						LIGGGHTS					
**Source**	**DF**	**Sum of Squares**	**Mean Squares**	**F-value**	***p*-value**	**Source**	**DF**	**Sum of Squares**	**Mean Squares**	**F-value**	***p*-value**
Model	5	50.644	10.13	27.811	0.0101	Model	5	140.74	28.15	23.205	0.0132
Linear	2	47.48	23.74	65.190	0.0034	Linear	2	138.197	69.10	5.982	0.0041
Square	1	1.756	1.7556	4.821	0.1156	Square	1	0.504	0.504	0.4157	0.5650
Interaction	2	1.408	0.7042	1.9338	0.2887	Interaction	2	2.039	1.019	0.8407	0.5130
Residuals	3	1.092	0.3642	–	–	Residuals	3	3.638	1.213	–	–
Lack-of-fit	3	1.092	0.3642	1	0.5	Lack-of-fit	3	3.638	1.213	1	0.5
Pure error	0	–	0	–	–	Pure error	0	0	0	–	–
Total	11	52.828	–	–	–	Total	11	148.016	–	–	–
$R^2 = 0.9789$		–	–	–	–	$R^2 = 0.9748$		–	–	–	–
Adj-$R^2 = 0.9437$		–	–	–	–	Adj-$R^2 = 0.9328$		–	–	–	–
**Term**		**Estimate**	**Std. Error**	**t-value**	***p*-value**	**Term**		**Estimate**	**Std. Error**	**t-value**	***p*-value**
Intercept		41.34556	0.44979	91.9216	<0.001	Intercept		40.62111	0.82079	49.4904	<0.001
×1		1.73833	0.24636	7.056	0.0059	×1		3.07667	0.44956	6.8437	0.0064
×2		−2.21167	0.24636	−8.9773	0.0029	×2		−3.68333	0.44956	−8.1931	0.0038
×1×2		−0.6625	0.30173	−2.1957	0.1156	×1×2		−0.355	0.5506	−0.6447	0.5650
$\times1^2$		−0.27833	0.42671	−0.6523	0.5607	$\times1^2$		−0.50667	0.77867	−0.6507	0.5616
$\times2^2$		0.79167	0.42671	1.8553	0.1606	$\times2^2$		0.87333	0.77867	1.1216	0.3437

×1 ~ (E - 2500)/1125; ×2 ~ (CED – 5×10^6)/$2 \cdot\times 10^6$

Table S18. ANOVA results of the quadratic model and estimated regression coefficients for SHAPE QUALITY of the COMPACT (Calibration).

EDEM						LIGGGHTS					
**Source**	**DF**	**Sum of Squares**	**Mean Squares**	**F-value**	***p*-value**	**Source**	**DF**	**Sum of Squares**	**Mean Squares**	**F-value**	***p*-value**
Model	5	3.66667	0.73	6.600	0.07564	Model	5	12.4444	2.49	4.200	0.1336
Linear	2	2.16667	1.08	9.750	0.0487	Linear	2	12.3333	6.17	10.406	0.0447
Square	1	1	1	9	0.0577	Square	1	0	0	0	1
Interaction	2	0.5	0.25	2.25	0.2530	Interaction	2	0.1111	0.556	0.0938	0.9131
Residuals	3	0.33333	0.11111	–	–	Residuals	3	1.7778	0.5926	–	–
Lack-of-fit	3	0.33333	0.11111	1	0.5	Lack-of-fit	3	1.7778	0.5926	1	0.5
Pure error	0	0	0	–	–	Pure error	0	0	0	–	–
Total	11	4	–	–	–	Total	11	16	–	–	–
R^2= 0.9167		–	–	–	–	R^2 = 0.8750		–	–	–	–
Adj-R^2 = 0.7778		–	–	–	–	Adj-R^2 = 0.6667		–	–	–	–

**Term**	**Estimate**	**Std. Error**	**t-value**	***p*-value**	**Term**	**Estimate**	**Std. Error**	**t-value**	***p*-value**
Intercept	5	0.24845	20.1246	<0.001	Intercept	3.77778	0.57378	6.5841	0.0071
×1	−0.33333	0.13608	−2.4495	0.0917	×1	−0.83333	0.31427	−2.6517	0.0769
×2	0.5	0.13608	3.6742	0.0349	×2	1.16667	0.31427	3.7123	0.0340
×1×2	0.5	0.16667	3.0000	0.0577	×1×2	0	0.38490	0.0000	1.0000
$\times1^2$	0	0.23570	0.0000	1.0000	$\times1^2$	−0.16667	0.54433	−0.3062	0.7795
$\times2^2$	−0.5	0.23570	−2.1213	0.1240	$\times2^2$	−0.16667	0.54330	−0.3062	0.7795

×1 ~ (E - 2500)/1125; ×2 ~ (CED - $5\cdot\times 10^6$)/$2\cdot\times 10^6$

© 2020 by the authors. Licensee MDPI, Basel, Switzerland. This article is an open access article distributed under the terms and conditions of the Creative Commons Attribution (CC BY) license (http://creativecommons.org/licenses/by/4.0/).
